# Therapeutic efficacy of thrombin-preconditioned mesenchymal stromal cell-derived extracellular vesicles on *Escherichia coli*-induced acute lung injury in mice

**DOI:** 10.1186/s12931-024-02908-w

**Published:** 2024-08-07

**Authors:** Yuna Bang, Sein Hwang, Young Eun Kim, Dong Kyung Sung, Misun Yang, So Yoon Ahn, Se In Sung, Kyeung Min Joo, Yun Sil Chang

**Affiliations:** 1 Cell and Gene Therapy Institute, Samsung Medical Center, Seoul, 06351 Republic of Korea; 2 Department of Health Sciences and Technology, SAIHST, Sungkyunkwan University, Seoul, 06351 Republic of Korea; 3 Department of Pediatrics, Samsung Medical Center, Sungkyunkwan University School of Medicine, Seoul, 06351 Republic of Korea; 4 Department of Anatomy & Cell Biology, Sungkyunkwan University School of Medicine, Suwon, 16419 Republic of Korea

**Keywords:** Acute lung injury, Mesenchymal stromal cells, Extracellular vesicles, *E. coli*

## Abstract

**Background:**

Acute lung injury (ALI) following pneumonia involves uncontrolled inflammation and tissue injury, leading to high mortality. We previously confirmed the significantly increased cargo content and extracellular vesicle (EV) production in thrombin-preconditioned human mesenchymal stromal cells (thMSCs) compared to those in naïve and other preconditioning methods. This study aimed to investigate the therapeutic efficacy of EVs derived from thMSCs in protecting against inflammation and tissue injury in an *Escherichia coli (E. coli)*-induced ALI mouse model.

**Methods:**

In vitro, RAW 264.7 cells were stimulated with 0.1 µg/mL liposaccharides (LPS) for 1 h, then were treated with either PBS (LPS Ctrl) or 5 × 10^7^ particles of thMSC-EVs (LPS + thMSC-EVs) for 24 h. Cells and media were harvested for flow cytometry and ELISA. In vivo, ICR mice were anesthetized, intubated, administered 2 × 10^7^ CFU/100 µl of *E. coli*. 50 min after, mice were then either administered 50 µL saline (ECS) or 1 × 10^9^ particles/50 µL of thMSC-EVs (EME). Three days later, the therapeutic efficacy of thMSC-EVs was assessed using extracted lung tissue, bronchoalveolar lavage fluid (BALF), and in vivo computed tomography scans. One-way analysis of variance with post-hoc TUKEY test was used to compare the experimental groups statistically.

**Results:**

In vitro, IL-1β, CCL-2, and MMP-9 levels were significantly lower in the LPS + thMSC-EVs group than in the LPS Ctrl group. The percentages of M1 macrophages in the normal control, LPS Ctrl, and LPS + thMSC-EV groups were 12.5, 98.4, and 65.9%, respectively. In vivo, the EME group exhibited significantly lower histological scores for alveolar congestion, hemorrhage, wall thickening, and leukocyte infiltration than the ECS group. The wet-dry ratio for the lungs was significantly lower in the EME group than in the ECS group. The BALF levels of CCL2, TNF-a, and IL-6 were significantly lower in the EME group than in the ECS group. In vivo CT analysis revealed a significantly lower percentage of damaged lungs in the EME group than in the ECS group.

**Conclusion:**

Intratracheal thMSC-EVs administration significantly reduced *E. coli*-induced inflammation and lung tissue damage. Overall, these results suggest therapeutically enhanced thMSC-EVs as a novel promising therapeutic option for ARDS/ALI.

**Supplementary Information:**

The online version contains supplementary material available at 10.1186/s12931-024-02908-w.

## Background

Acute respiratory distress syndrome (ARDS) and severe acute lung injury (ALI) are critical respiratory diseases characterized by uncontrolled inflammation, bilateral lung damage, fibrosis, and non-cardiogenic pulmonary edema [1–3]. ARDS/ALI develops by primary causes including bacterial or viral pneumonia, inhalation of toxic substances, or sepsis, and leads to poor prognosis and high mortality rates [2, 4, 5]. Treatment strategies for ARDS/ALI traditionally involve combinations of antibiotics and prone positioning to address individual symptoms without any definite treatment options. Considering the severity and diverse complications, such as inflammation, edema, and fibrosis, a comprehensive therapeutic approach to improve the overall pathophysiology of ALI is urgently needed [6, 7].

Recently, new therapeutic modalities for ARDS/ALI are being explored using mesenchymal stromal cells (MSCs) or MSC-derived extracellular vehicles (EVs) [8–10]. The therapeutic efficacy of MSCs depends on paracrine signaling, with bioactive factors released into EVs [11–13]. Paracrine enhancement of MSCs through various priming methods has shown promise because EV cargo content is stimulus-dependent [14, 15]. Our previous investigations revealed that EV production and cargo content significantly increase, primarily via proteinase-activated receptor (PAR)-1 and partly via a PAR3-dependent pathway, in thrombin-preconditioned human MSCs (thMSCs) compared to those in naïve and other preconditioning methods [16, 17]. Furthermore, the transplantation of thMSCs in neonatal rat models of intraventricular hemorrhage and hypoxic-ischemic encephalopathy, as well as thMSC-derived EVs in neonatal meningitis, confirmed their significant therapeutic efficacy in attenuating inflammation, decreasing cell death, and reducing subsequent tissue injuries [18–20].

Among the pathophysiological processes of ALI, inflammation, leukocyte infiltration, impaired vascular permeability, edema, and fibrosis are closely associated with PAR signaling, a member of the G protein-coupled receptor family expressed in epithelial, endothelial, and immune cells [21, 22]. On inflammation and tissue damage, increased thrombin production cleaves and activates PARs triggering a cascade of reactions, leading to the release of prothrombotic mediators, inflammatory cytokines, and chemokines IL-6, TNF-α, and CCL2 [22]. This cascade increases vascular permeability, endothelial activation, and edema, ultimately causing severe tissue injury [23–25] .

Thus, we hypothesized that EVs derived from MSCs with enhanced function via thrombin preconditioning-mediated PAR activation (thMSC-EVs) would provide substantial protection against ARDS/ALI [17]. This study aimed to assess the therapeutic efficacy of EVs from thrombin-preconditioned Warton jelly-derived MSCs (thWJ-MSCs) in an *Escherichia coli (E. coli)*-induced ALI mouse model. To our knowledge, this is the first investigation of thrombin-preconditioned MSC-derived EVs in an ARDS/ALI preclinical model.

## Methods

### WJ-derived MSCs preparation

Human WJ-MSCs were provided by the Good Manufacturing Practice Facility of Samsung Medical Center and expanded as previously described [20]; WJ-MSCs from passage 6 were used in this study and were characterized of its surface markers, proliferation rate, and differentiation potential according to the minimal MSC criteria set by the ISCT (Supplementary Figure S1). WJ-MSCs were cultured in minimum essential medium (MEM)-α (Gibco; Grand Island, NY, USA) with 10% fetal bovine serum (FBS. Gibco; Grand Island, NY, USA) and 0.1% gentamicin (Gibco; Grand Island, NY, USA) in a 5% CO_2_ humidified incubator at 37 ℃. Thrombin preconditioning was done following the previously established method [20]. Briefly, At 90% confluency, the culture medium was washed three times with Dulbecco’s phosphate-buffered saline (Welgene; Daegu, South Korea) to remove residual FBS, and replaced with serum-free MEMα supplemented with 20 units/mL of thrombin (Reyon Pharmaceutical Co, Ltd; Seoul, South Korea) for 3 h. The levels of HGF and VEGF in thrombin-preconditioned WJ-MSCs measured from the conditioned medium are presented in Supplementary Figure S2.

### EV isolation & quantification

**Fig. 1:**
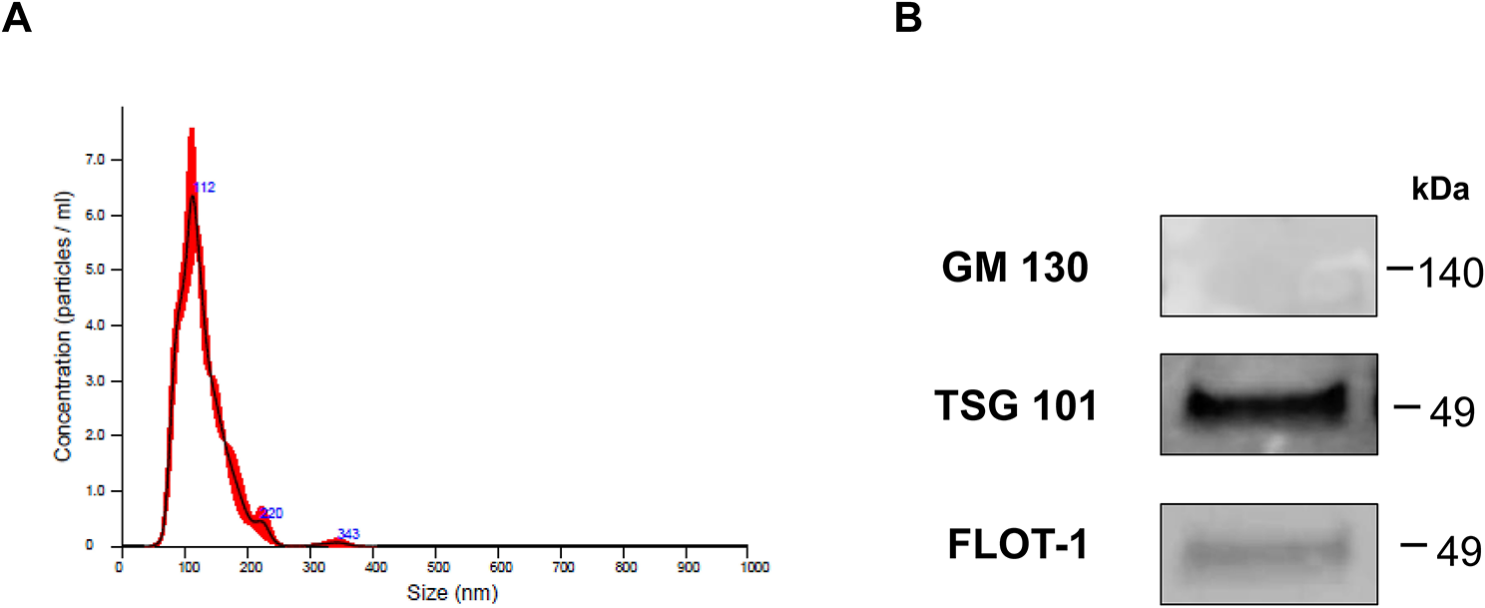
Characterization of thrombin preconditioned WJ-MSCs-derived EVs. (**A**) Nanoparticle tracking analysis (NTA) evaluated protein concentration and size distribution. (**B**) EV-specific markers were analyzed using western blot. GM130, negative EV marker (Golgi membrane marker); TGS101, and Flotillin-1 are positive markers of EV surface. Full blot images can be found in Figure S9. GM130, Golgi matrix protein 130; TGS101, Tumor susceptibility gene 101; FLOT-1, Flotillin-1

The thrombin preconditioned medium of WJ-MSCs was harvested and filtered using a 0.2 μm bottle top vacuum filtration system (Corning; Corning, NY, USA). EVs were then isolated and diafiltrated in DPBS using a tangential flow filtration system (KrosFlo^®^ KR2i, Repligen; Waltham, MA, USA) with pore size 300 kDa mPES membrane (S02-E300-05-N, Repligen; Waltham, MA, USA). Subsequently, the concentrated EVs were filtered via a 0.2 μm filter (S6534-FMOSK, Sartorius; Göttingen, Germany) and analyzed using Nanoparticle Tracking Analysis (NanoSight NS300; Malvern, Malvern, UK) (Fig. 1). thMSC-EVs were aliquoted and stored at -70 ℃ until subsequent experiments. thMSC-EVs were confirmed of markers GM130 (1:1000; Cell Signaling Technology, Danvers, MA, USA), TSG101 (1:1000; Abcam, Cambridge, UK), and flotillin-1 (1:1000; Cell Signaling Technology, Danvers, MA, USA) using western blot. The size of naïve MSC-EVs are presented in Supplementary Figure S3.

### *E. Coli* preparation

Kanamycin-resistant *E. coli* strain E69 was kindly provided by Dr. Kwang Sik Kim from Johns Hopkins Hospital. The *E. coli* was cultured in suspension overnight in Brain-Heart-Infusion broth (BHI, BD Bioscience; Franklin Lakes, NJ, USA) with 53 µg/mL kanamycin (Sigma Aldrich; Burlington, Massachusetts, USA) at 37 °C and 200 rpm. 300 µL of cultured broth was freshly diluted in 7 mL BHI broth, further incubated for 2 h, and centrifuged for 7 min at 3500 rpm. Optical density (OD) was measured at 600 nm and diluted to an OD value of approximately 0.6 using a Multiskan Sky spectrophotometer (Thermo Fisher Scientific, Waltham, MA, USA). 100 µl of *E. coli* culture was then spread on BHI agar plates using a sterilized spreader (SPL Life Science, Pocheon-si, South Korea) and incubated the plates overnight at 37 °C. The final *E. coli* concentration in 50 µL normal saline was 10^7^ colony-forming units (CFU).

### In vitro LPS-induced ALI inflammation modeling using alveolar macrophages

Alveolar macrophage cell line, RAW 264.7 (Korean Cell Line Bank; Seoul, Republic of Korea), were maintained in Dulbecco’s Modified Eagle Medium (Gibco; Grand Island, NY, USA) supplemented with 10% FBS and 1% penicillin/streptomycin (Invitrogen, Carlsbad, California, USA) in a humidified chamber under 5% CO_2_ at 37 ℃, as previously described [26]. At 80% confluence, RAW264.7 cells were stimulated with 0.1 µg/mL lipopolysaccharide (LPS O111:B4. Sigma Aldrich; Burlington, Massachusetts, USA) for 1 h in a 96-well plate. Then, equal volumes (5 µL) of PBS and thMSC-EVs (5 × 10^7^ particles / 5 µL) were added as the LPS Ctrl and LPS + thMSC-EVs groups, respectively, and maintained for 24 h. Culture media were collected for Enzyme-linked immunosorbent assay (ELISA) of pro-inflammatory cytokines.

### Flow cytometric analysis

The extent of M1 activation and M2 activation in RAW 264.7 cells was assessed using flow cytometry. RAW 264.7 cells in the normal control (NC), LPS Ctrl, and LPS + thMSC-EV groups were collected and centrifuged at 450 × *g* at 4 °C for 10 min. Anti-CD86 antibody (BD Biosciences, Franklin Lakes, NJ, USA) and anti-CD206 antibody (BD Biosciences, Franklin Lakes, NJ, USA) were incubated for 20 min and FACS was performed as previously described [27]. Dead cells and doublets were excluded from the population.

### ALI animal model

**Fig. 2:**
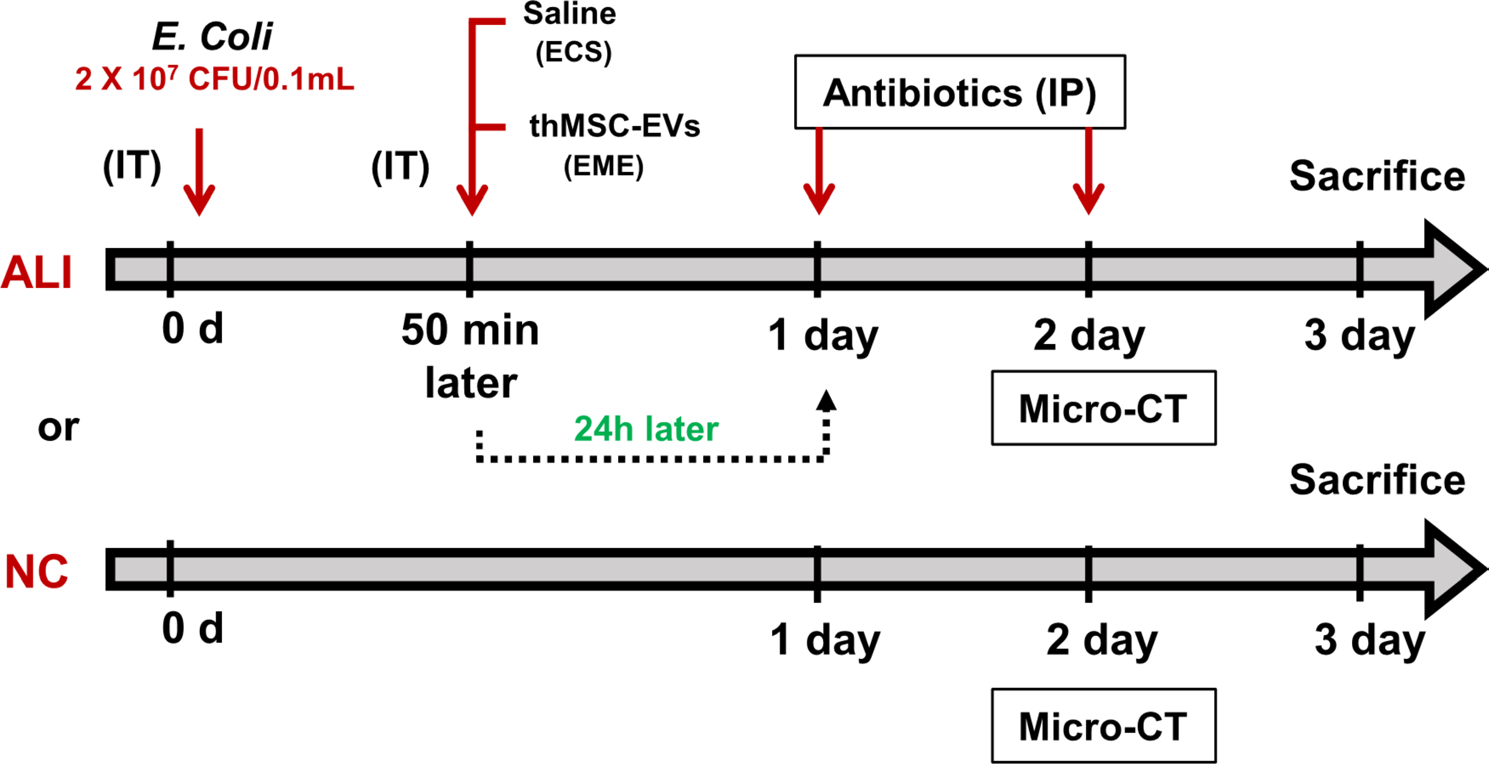
In vivo experimental design. ALI, acute lung injury; NC, normal control; *E. coli*,* Escherichia coli;* thMSC-EVs, EVs derived from thrombin-preconditioned Warton’s jelly MSCs; IP, intraperitoneal injection; IT, intratracheal injection; Micro CT, Micro-computed tomography

All animal experimental protocols were reviewed and approved by the Institutional Animal Care and Use Committee (IACUC, Approval number: 20,230,126,004) of the Samsung Biomedical Research Institute, an AAALAC International (Association for Assessment and Accreditation of Laboratory Animal Care)-accredited facility, under the National Institutes of Health Guidelines for Laboratory Animal Care. A brief description of the experimental design is presented in Fig. 2. 8 weeks old ICR male mice were purchased from Orient Co. (Seoul, Republic of Korea) and stabilized for 1 week. Experimental ALI induction was performed as shown in Fig. 2. The mice were anesthetized using an intraperitoneal (IP) injection of 45 mg/kg ketamine and 8 mg/kg xylazine cocktail. The vocal cords of the mice were visualized using an otoscope by placing the animals in an inclined plane, as previously described [28]. Mice were endotracheally intubated using a catheter (introcan certo catheter 22G, 0.9 $\:\times\:$ 25 mm, B/Braun, Melsungen, Germany). *E. coli* (2 × 10^7^ CFU/100 µL) was administered into the lungs of mice via the catheter. 50 min after *E. coli* administration, the ALI control (ECS) and thMSC-EVs treated (EME) groups were administered equal volumes of saline (50 µL) and thMSC-EVs (1 × 10^9^ particles/50 µL), respectively. The timing of administration was determined based on time-dependent cytokine and bacterial CFU measurements (Supplementary Figure S5, Supplementary Table 1). The survival and body weight of the mice were monitored daily (Supplementary Figure S6). Ceftriaxone (100 mg/kg) was administered IP once daily. In vivo micro-computed tomography (micro-CT) scanning of the lungs was performed two days post-injury. On day 3, the mice were anesthetized with pentobarbital (60 mg/kg, IP) to collect the lung tissues and bronchoalveolar lavage fluid (BALF) for further analysis. Transcardial perfusion was performed prior to tissue excision. The excised lungs were then inflated with saline using a 3 mL syringe, fixed overnight in 4% paraformaldehyde, and embedded in a paraffin block. BALF was collected by irrigating twice with 1 mL of aseptic saline using a 22-gauge catheter, as previously described [26].

### Micro CT imaging and lung injury analysis

Micro CT scanning was performed using the Siemens Inveon Micro-PET/CT scanner (Siemens Medical Solutions, Knoxville, TN, USA). All micro CT analysis protocol and figures are presented in Table S2, Figure S7, and Figure F8 under the Supplementary File. The mice were anesthetized with 2–3% isoflurane in 100% oxygen during the scan. The CT images were obtained during the expiratory breathing phase. Briefly, each mouse was scanned for 20 min using a 1.5 mm-thick aluminum filter. For each scan, the Inveon Acquisition Workplace (IAW, Siemens Medical Solutions, Knoxville, TN, USA) software package was used to reconstruct the data into an effective pixel size with a downsampling factor of 2 using the Shepp and Logan filter back-projection algorithm. A phantom scan was conducted for Hounsfield unit (HU) calibration, establishing grayscale values for air and water (ranging from 0 to -1000 HU).

Ten axial micro-CT slices were selected for tissue analysis. The criteria for slide selection were strictly applied to all animals to reduce inter-animal variance. The first slide without a visible diaphragm was marked as the first slide. Every eighth image and ten consecutive images were selected. All images for analysis were matched for 24-bit and the same field of view was used to remove variance. The aerated regions of the lung were semi-automatically measured using the Inveon Research Workplace (IRW, Siemens Medical Solutions, Knoxville, TN, USA) software. The tissue regions of the lungs, excluding the heart, were manually outlined by the investigator using the ImageJ software (National Institutes of Health, Bethesda, MD, USA). The detailed methods are provided in the Supplementary Material. The percentage of damaged tissue was calculated using the following equation:

$$\eqalign{& \% Damaged\,\,Tissue = \cr & {{\left( {Whole\,\,lung\,\,volume} \right) - \left( {Aerated\,tissue\,volume} \right)} \over {Whole\,\,lung\,\,volume}} \times 100\left( \% \right) \cr}$$

All analyses were performed blindly.

### Lung injury scores

Paraffin-embedded lung tissues were sectioned into 5 μm-thick slices, deparaffinized, and stained using hematoxylin and eosin (H&E). Three slides per tissue representing the ventral, medial, and dorsal regions of the lungs were selected for analysis. Furthermore, a total of 24 serial images, 8 images from the left lobe and 16 images from the right lobe, were taken and scored. Histological lung injury scores were measured using the following four criteria specified in a previous study [28]: alveolar congestion, alveolar hemorrhage, infiltration of leukocytes, and thickening of the alveolar wall. Each category was scored on a five-point scale from 0 to 4 as follows: 0, no or minimal lung injury and 1, 2, 3, and 4 with lung injury in 25, 50, 75 and > 75% of the field, respectively. All data were analyzed blindly.

### Wet-dry lung ratios

To assess pulmonary edema, the wet weight of the lungs was measured immediately after excision. Subsequently, the lungs were dried at 60 ℃ for 72 h to measure the dry weight. The wet/dry ratio for each lung was assessed by dividing the mass of the wet lung by that of the dry lung, as described previously [28].

### ELISA for inflammatory cytokines

To assess the levels of pro-inflammatory cytokines, such as CCL-2, IL-1α, IL-1β, IL-6, TNF-α, and Interferon Gamma (IFN-γ) in the lungs, commercial ELISA kits (R&D Systems, Minneapolis, MN, USA) were used following the manufacturer’s instructions.

### Statistical analyses

All data were analyzed using GraphPad Prime 8 software (GraphPad, San Diego, CA, USA). Survival curves were assessed using the log-rank test. One-way analysis of variance with post-hoc Tukey’s test was used to statistically compare the experimental groups. Data in bar graphs are presented with mean ± standard error of mean (SEM). In the box and whisker plots, the first, median, and third quartiles are presented as boxes, and the minimum and maximum are presented as whiskers. Specific p values and sample populations are indicated in the figure legends. Statistical significance was set at *p* < 0.05.

## Results

### thMSC-EVs significantly reduced M1 polarization and pro-inflammatory cytokine secretions of LPS-induced RAW 264.7 cells

**Fig. 3:**
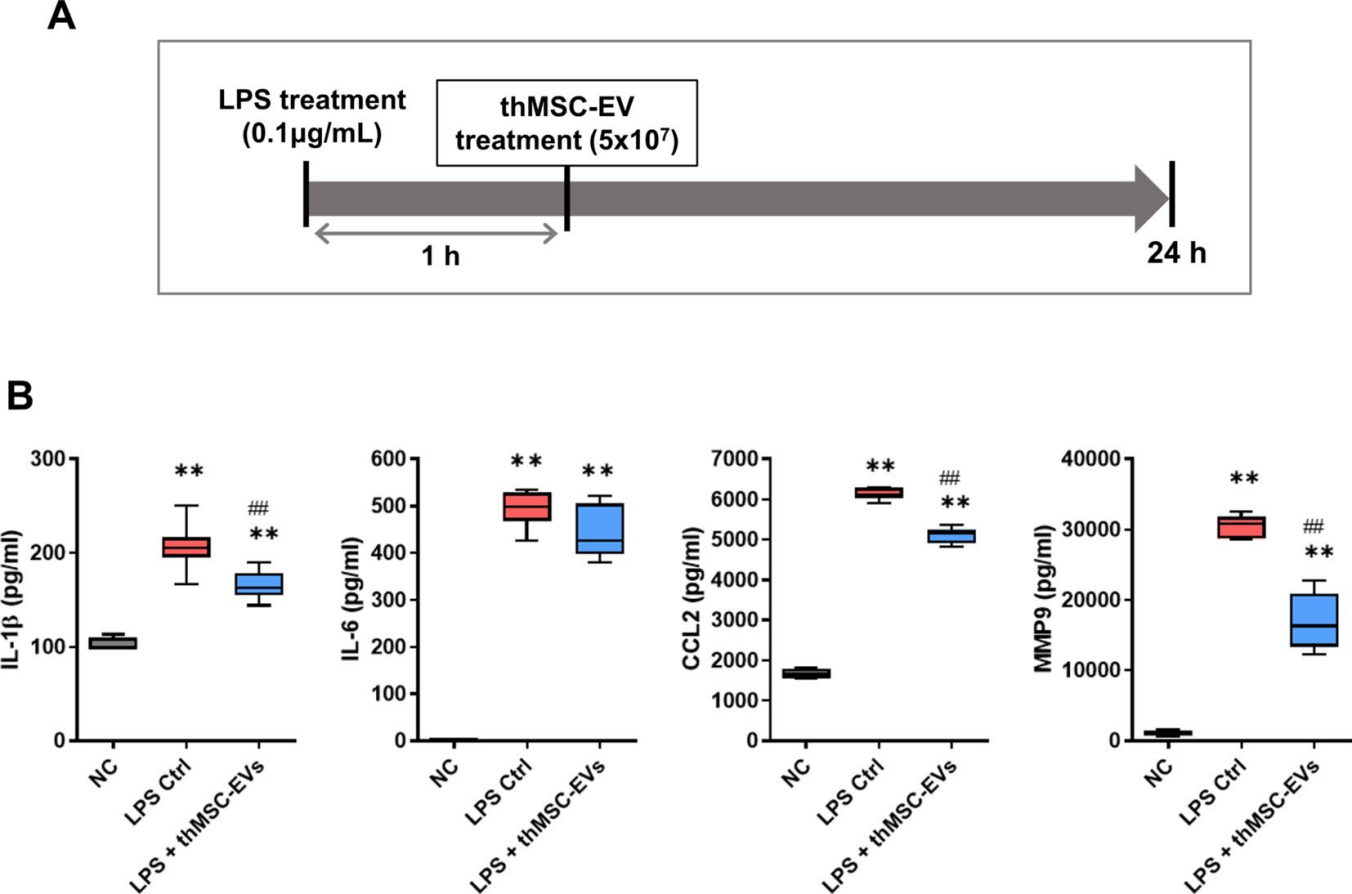
Anti-inflammatory effect of thMSC-EVs in LPS-stimulated RAW 264.7 cells. (**A**) Study design of the LPS-stimulated RAW 264.7 cells ALI in vitro model. (**B**) The levels of pro-inflammatory cytokines IL-1β, IL-6, CCL-2, and MMP-9 were measured using ELISA. *n* = 4, 6, 6 in the NC, LPS Ctrl, and LPS + thMSC-EVs groups, respectively. Data are presented as box and whisker plot. whiskers represent the min and max. **, *p* < 0.01 vs. NC; ##, *p* < 0.01 vs. LPS Ctrl group. One-way analysis of variance (ANOVA) post hoc Tukey analysis was used. NC, normal control; LPS Ctrl, LPS control group; LPS + thMSC-EVs, thMSC-EVs treated group

**Fig. 4:**
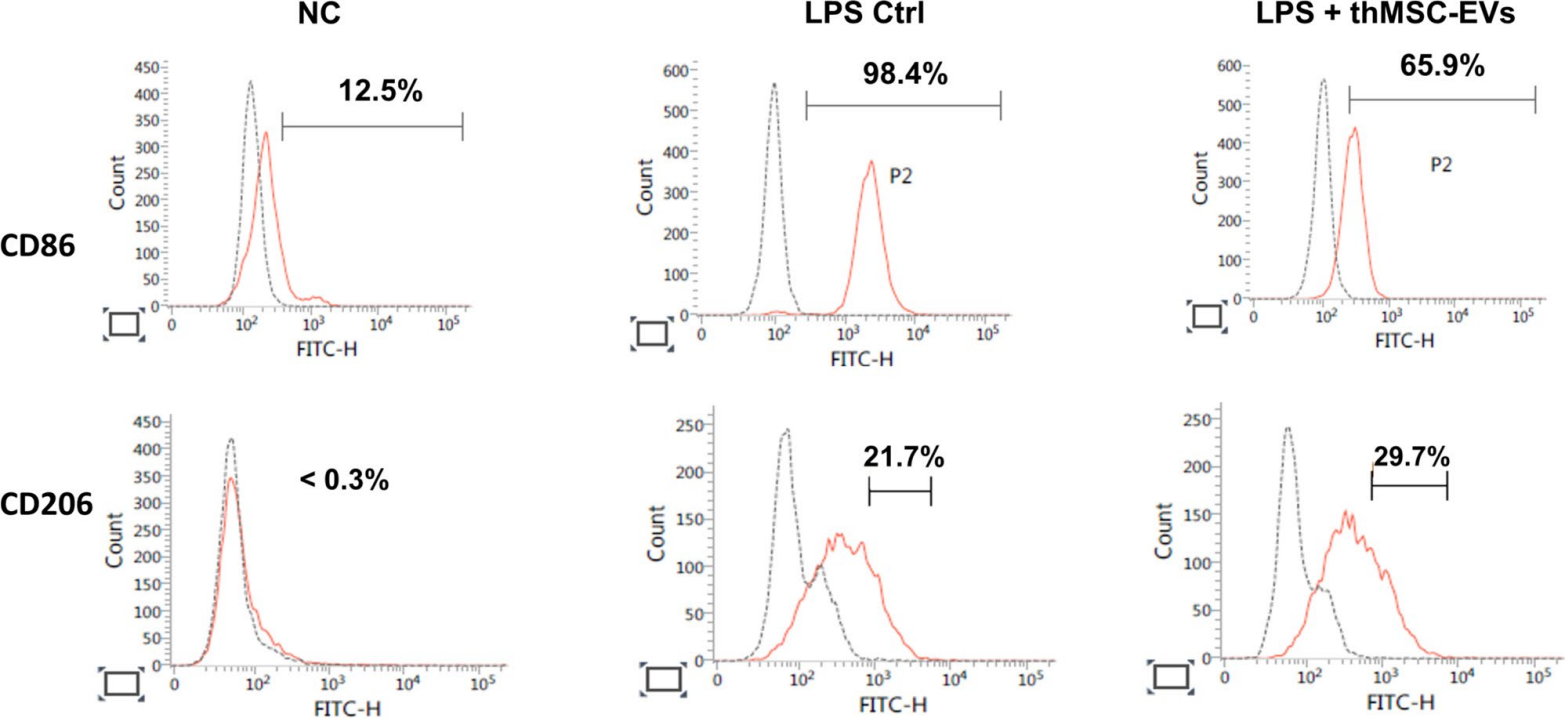
Flow cytometric analysis of LPS-induced RAW 264.7 cells. Percent representation of CD86 (a marker of M1 macrophage) and CD206 (a marker of M2 macrophage) expressing RAW 264.7 cells. NC, normal control; LPS Ctrl, LPS control group; LPS + thMSC-EVs, thMSC-EVs treated group

LPS-induced mouse alveolar macrophage RAW 264.7 cells were treated with either thMSC-EVs or PBS after 1 h of LPS stimulation (Fig. 3A). The levels of inflammatory cytokines (IL-1β, IL-6, and CCL-2) and MMP-9 and the extent of M1 macrophage polarization were measured 24 h after (Figs. 3B and 4) using ELISA and flow cytometry, respectively. The levels of IL-1β, IL-6, CCL-2, and MMP-9 were significantly higher in the LPS Ctrl and LPS + thMSC-EVs groups than those in the NC group. LPS + thMSC-EVs group was significantly lower in the levels of IL-1β, CCL-2, and MMP-9 compared to the LPS Ctrl group, however, the level of IL-6 did not reach statistical significance. FACS confirmed that the percentages of M1 polarization in the normal control, LPS Ctrl, and LPS + thMSC-EV groups were 12.5, 98.4, and 65.9%, respectively (Fig. 4). The percentages of M2 polarization in the normal control, LPS Ctrl, and LPS + thMSC-EV groups were < 0.3%, 21.7%, and 29.7%, respectively. Dead cells and doublets were excluded from the population (Supplementary Figure S4).

### Intratracheal administration of thMSC-EVs attenuated tissue injury in *E. Coli*-induced ALI mouse

**Fig. 5:**
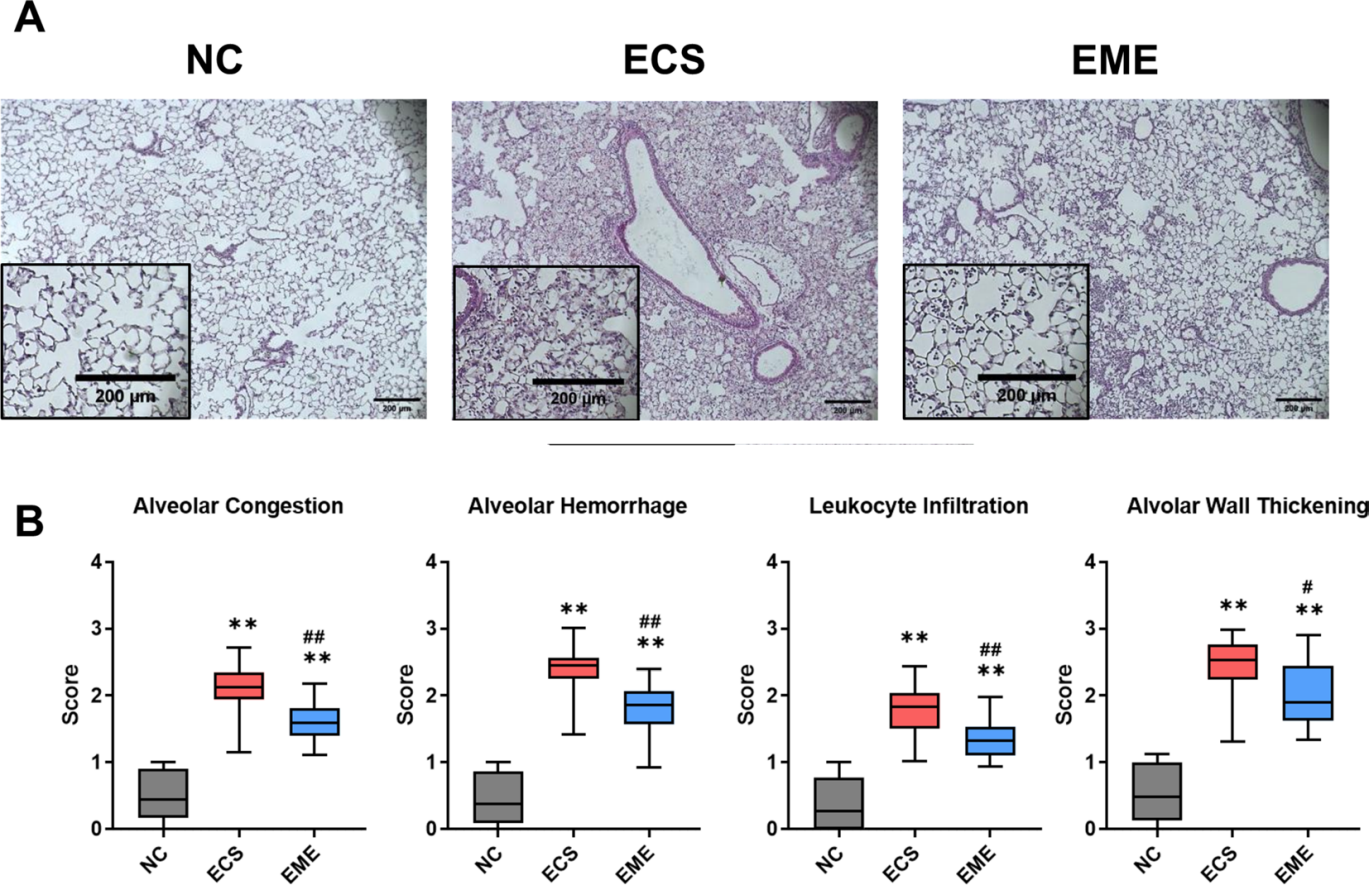
Intratracheal administration of thMSC-EVs attenuated tissue injury in *E. coli*-induced ALI mice. (**A**) Representative microscopic images of lung tissues of each group. (Original magnification;$\:\:\times\:\:$40, scale bars; 200 μm) (**B**) Scored histological grades of alveolar congestion, alveolar hemorrhage, leukocyte infiltration, and alveolar wall thickening in lung tissue. *n* = 10, 14, and 19 in NC, ECS, and EME groups, respectively. Data are presented as a box and whisker plot. Whiskers represent the min and max. **, *p* < 0.01 vs. NC; #, *p* < 0.05 vs. ECS; ##, *p* < 0.01 vs. ECS. One-way ANOVA post hoc Tukey was used. NC, normal control; ECS, *E. coli*-induced ALI control group; EME, thMSC-EVs treatment group after *E. coli*-induced ALI

The lungs of *E. coli*-induced ALI mice were excised, fixed, paraffin-embedded, and sectioned for histological evaluation. Figure 5A shows the representative H&E-stained lung sections from each group. Histologic scores of ALI pathophysiology including alveolar congestions, hemorrhage, wall thickening, and leukocyte infiltration were higher in the *E.coli-*induced ALI lung tissues than in the NC group (Fig. 5B). These elevated scores were significantly reduced after thMSC-EV administration, as evidenced by the significantly lower scores in the EME group than those in the ECS group.

### Intratracheal thMSC-EV administration attenuated pulmonary edema in *E. Coli*-induced ALI mouse

**Fig. 6:**
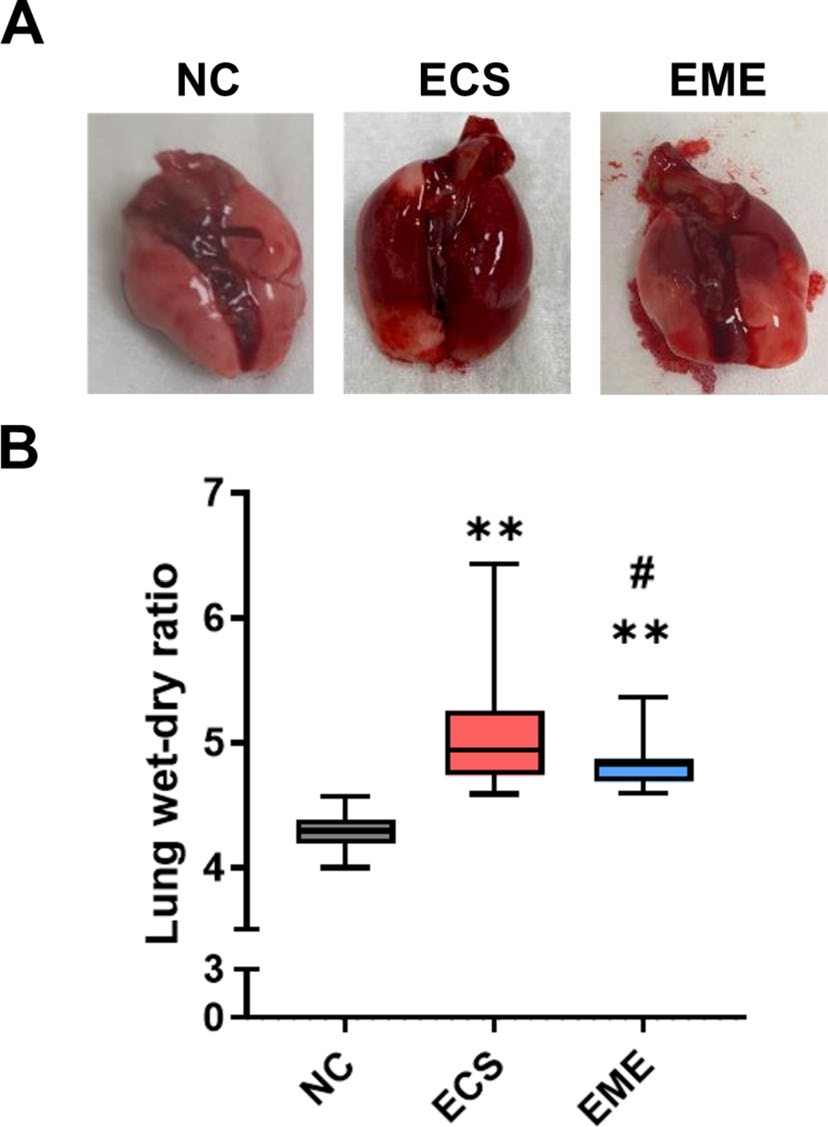
Lung tissue and lung water content. (**A**) The lung tissue of NC, ECS, and EME groups. (**B**) Lung water content was measured as wet-dry lung ratio in normal control, ALI control, and treated with the thMSC-EVs group. *n* = 14, 19, and 23 in the NC, ECS, and EME groups, respectively. Data are presented as a box and whisker plot. Whiskers represent the min and max. **, *p* < 0.01 vs. NC; #, *p* < 0.05 vs. ECS. One-way ANOVA post hoc Tukey was used. NC, normal control; ECS, *E. coli*-induced ALI control group; EME, thMSC-EVs treatment group after *E. coli*-induced ALI

The extent of *E. coli* -induced pulmonary edema was evaluated by measuring the tissue wet-dry mass ratio. Representative images of tissues are shown in Fig. 6A. A higher wet-dry ratio indicates more fluid in the lung tissue. *E. coli* induction significantly increased the wet-dry ratio, in both the ECS and EME groups compared to that in the NC group (Fig. 6B). However, this ratio was significantly lower in the EME group than in the ECS group.

### thMSC-EVs attenuated inflammatory cytokine levels in the BALF of *E. Coli*-induced ALI mouse

**Fig. 7:**
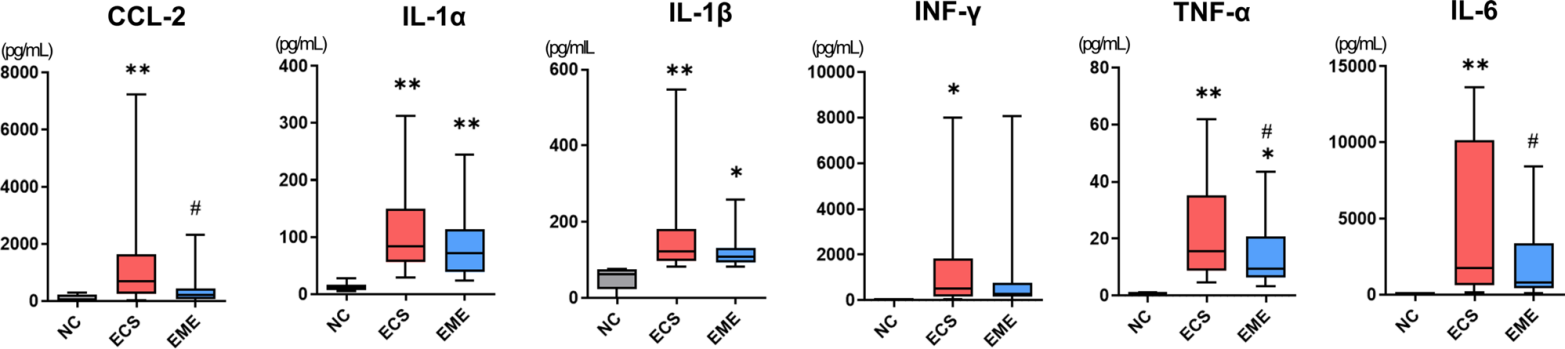
Intratracheal administration of thMSC-EVs attenuated inflammatory cytokine secretion in *E. coli*-induced ALI mice. The levels of pro-inflammatory cytokines such as CCL-2, IL-1α, IL-1β, INF-γ, TNF-α, and IL-6 were measured using ELISA. *n* = 18, 25, and 25 in the NC, ECS, and EME groups, respectively. Data are presented as a box and whisker plot. Whiskers represent the min and max. **, *p* < 0.01 vs. NC; *, *p* < 0.05 vs. NC; #, *p* < 0.05 vs. ECS. One-way ANOVA post hoc Tukey analysis was used. NC, normal control; ECS, *E. coli*-induced ALI control group; EME, thMSC-EVs treatment group after *E. coli*-induced ALI

The obtained BALF samples were used for cytokine analysis. Levels of the pro-inflammatory cytokines, CCL-2, IL-1α, IL-1β, IL-6, TNF-α, and IFN-γ were measured (Fig. 7). These were significantly higher in the ECS group than in the NC group. Only IL-1α, IL-1β, and TNF-α levels were significantly increased in the thMSC-EVs-treated EME group compared to those in the NC group. The levels of CCL2, TNF-α, and IL-6 in the EME group were significantly lower than those in the ECS group. Moreover, the levels of CCL2, IFN-γ, and IL-6 were not significantly different from those in the NC group. The levels of IL-1α and IL-1β in the EME group were not significantly different from those in the ECS group, however, the mean value was lower in the EME group.

### CT analysis revealed that thMSC-EVs significantly attenuated lung damage in *E. Coli*-induced ALI mouse

**Fig. 8:**
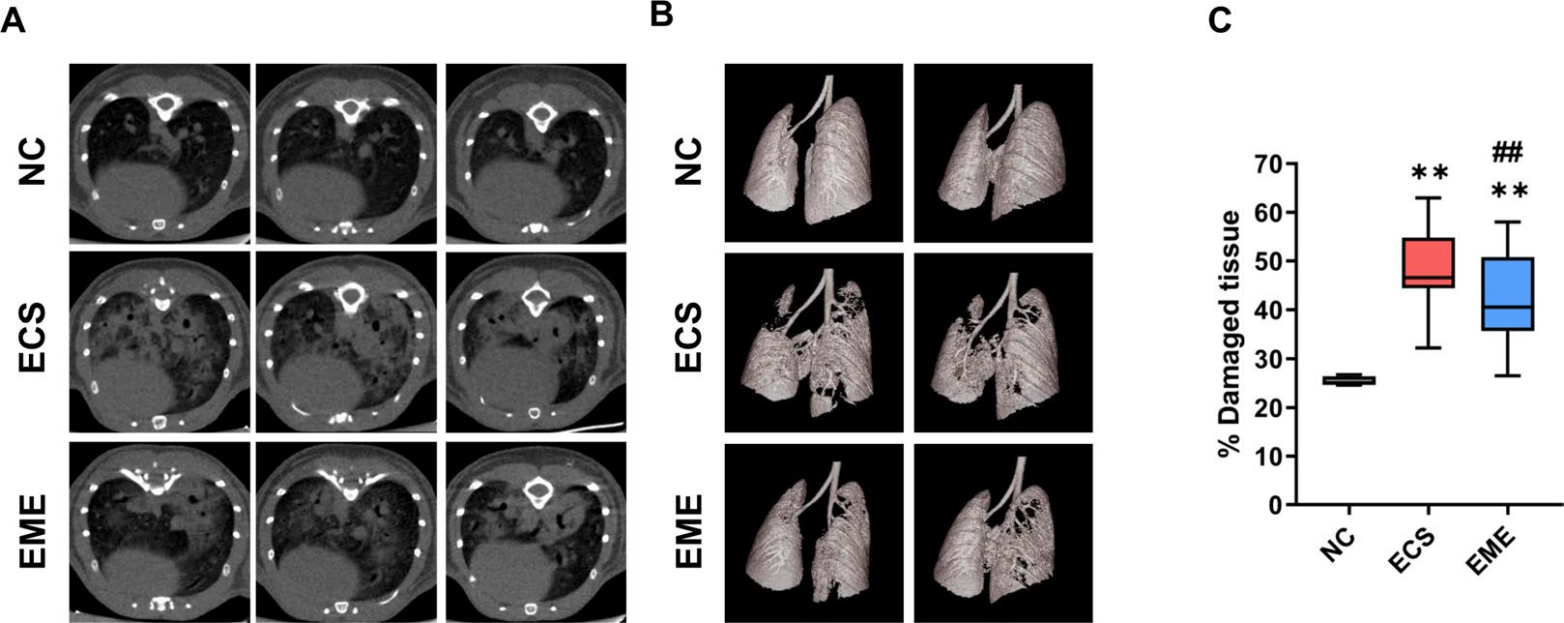
Computerized tomography (CT) scans of mice lungs. (**A**) The air-filled areas in the mice lung CT scans appeared as dark backgrounds, while the lesions resulting from the *E. coli* administration were observed as hyperintense patches. (**B**) Semi-automated 3D rendered image of the mouse lung parenchyma and airways. The white region represents the aerated parenchyma, allowing visualization of retained parenchyma. (**C**) Calculated percent of damaged lung using CT images from Fig. 8A. *n* = 4, 23, and 21 in the NC, ECS, and EME groups, respectively. Data are represented as a box and whisker plot. Whiskers represent the min and max. **, *p* < 0.01 vs. NC; ##, *p* < 0.01 vs. ECS. One-way ANOVA post hoc Tukey analysis was used. NC, normal control; ECS, *E. coli*-induced ALI control group; EME, thMSC-EVs treatment group after *E. coli*-induced ALI

In vivo, micro-CT was performed on each mouse to assess lung damage using the calculation of the percentage of damaged lung regions (Fig. 8A). The HU setting allows air-exchanging lung parenchyma to be distinguished from denser tissue areas such as infected lesions and injured tissue areas. The aerated lung parenchyma appeared dark, whereas lesions appeared as white patches. These images were used to generate a 3D-rendered aerated parenchyma (Fig. 8B) and calculate the percentage of damaged tissue (Fig. 8C). In Fig. 8B, only the aerated tissue regions appeared white, allowing visualization of the retained air-exchanging parenchyma. The percentage of damaged tissue in both *E. coli*-induced ECS and EME groups were significantly higher than that in the NC group. However, thMSC-EVs administration significantly reduced the percentage of damaged lungs in the EME group compared with that in the ECS group (Fig. 8C).

## Discussion

In the present study, we have demonstrated that intratracheal thMSC-EVs administration significantly attenuated lung injury in *E. coli* -induced ALI mice, as evidenced by decreased lung edema and inflammatory cytokine levels in the BALF, as well as decreased histological lung injury scores and damaged regions observed in lung CT. Numerous studies have investigated the therapeutic effects of MSC-EVs in ALI animal models [8–10] and proposed MSC-EVs as promising therapeutic candidates for ALI owing to their cell-free nature and low immunogenicity [29, 30]. However, enhancing the regenerative and protective potency of MSCs is critical, considering their severity, which leads to a high mortality rate. We have previously investigated the enhanced therapeutic efficacy of preconditioned MSCs, specifically with LPS and thrombin [26, 27, 31]. Preconditioning involves educating MSCs before transplantation to the injured area by pre-exposing them to specific stimuli, such as LPS or *E. coli* for inflammation and thrombin for hemorrhagic injury, allowing MSCs to readily exert therapeutic effects. *E. coli*-preconditioned MSCs exert anti-inflammatory and bactericidal effects by secreting defensin [31], whereas thrombin-preconditioned MSCs significantly increase angiogenic factors which improve vascular permeability and tissue injury through PAR1 and PAR3 signaling [17]. We have further confirmed the enhanced therapeutic efficacy of thMSCs compared to the naïve MSCs in neonatal IVH study, and further confirmed equivalent therapeutic efficacy of thMSCs and thMSC-EVs in neonatal meningitis study [18, 32]. Building on our previous confirmation of antimicrobial, anti-inflammatory, and anti-apoptotic effects of LPS-preconditioned MSCs in an *E. coli*-induced ALI mouse model [26, 31], this study aimed to further assess the therapeutic efficacy of thrombin-preconditioned MSCs derived EVs in tissue injuries using the same *E. coli*-induced ALI mouse model in the present study.

The interplay between inflammation and coagulation pathways mediating PAR activation in ARDS/ALI is well known to induce severe and diffuse lung tissue injury [2, 22, 23, 25, 33, 34]. Among the four PAR subtypes, PAR1, PAR3, and PAR4 are activated by thrombin, a serine protease that regulates blood coagulation. Hemorrhage increases thrombin levels in the area, further activating PAR-expressing endothelial, epithelial, and immune cells. Upon infection, activated immune cells induce vascular permeability, recruiting immune cells and blood coagulation factors to the injury site. Hypercoagulability can increase circulatory levels of fibrinogen and d-dimer, not only within the blood but also in the lungs [35]. Fibrin formation, reflecting localized microthrombi and endothelial damage in the pulmonary microcirculation, leads to plasma exudation, tissue factor-mediated thrombin generation, and the development of fibrinous hyaline membranes, a characteristic of the inflammatory response in ARDS [24]. The interaction between inflammation and vascular activation cumulatively induces lung damage [36]. An increase in PAR signaling has been recapitulated in multiple experimental ALI animal models [25, 37]. The present study observed that MSCs preconditioned with thrombin, a substance indicative of coagulative status, improved lung injury in E. coli-induced infectious ARDS via an anti-inflammatory response from EVs. We postulate that the protective factors secreted through EVs in PAR-activated thrombin-preconditioned MSCs may have been the mechanism of thMSC-EVs’ improvement of tissue injuries, which is known to be mediated by PAR. However, further study is needed to determine whether its efficacy varies according to the degree of hypercoagulable status in vivo, as evidenced by the variable level of hypercoagulability markers such as fibrinogen and d-dimer.

In this study, we histologically confirmed a significant reduction in leukocyte infiltration, pulmonary edema, and alveolar wall thickening after the intratracheal administration of thMSC-EVs. Our previous report demonstrated the thrombin-activated PARs in MSCs enhanced the secretion of the angiogenic cargo angiogenin, angiopoietin-1, and vascular endothelial growth factor (VEGF) in thrombin-preconditioned MSCs compared to those in LPS-preconditioned MSCs [17]. We have confirmed a significant increase in the secretion of HGF and VEGF in thrombin-preconditioned MSCs, in which the thMSC-EVs were isolated, compared to the naïve MSCs (Supplementary Figure S2). HGF, VEGF, angiogenin, and angiopoietin-1 are known to reduce inflammation, endothelial cell activation, apoptosis, and fibrosis [38–44], which presumably contributed to attenuating tissue injuries in the present study. Gupta et al. demonstrated the therapeutic effect of MSCs against bacterial pneumonia in a mouse model of PAR1-mutated mouse bone marrow-derived MSCs, suggesting that PAR signaling is critical for the survival and therapeutic efficacy of MSCs [45]. Our findings of reduced alveolar wall thickness, alveolar congestion, and leukocyte infiltration can be attributed to the protective cargo content of thMSC-EVs.

Upon thrombin activation, PAR-expressing epithelial, endothelial, and immune cells secrete significant levels of inflammatory cytokines and chemokines [22]. Classically activated M1 macrophages upregulate the production of pro-inflammatory cytokines, including IL-6, IL-1α, IL-1β, and TNF-α, which, under excessive secretion and accumulation, leads to increased vascular permeability and damaged alveolar epithelium and endothelium [2, 22, 26, 46, 47]. In this study, a significant reduction of pro-inflammatory cytokines IL-1β, CCL-2, and MMP-9 can be attributed to the suppression of M1 polarization, without meaningful modulation of M2 polarization, in LPS-induced RAW 264.7 cells (Fig. 4). Several studies have also reported a reduction of lung injury via M1 suppression, supporting the idea that macrophage polarization is a dynamic process rather than strictly dichotomous [48–51]. Decreased levels of the major neutrophil-recruiting factor, CCL-2, were also evident in BALF and tissue histology measurements. MMP-9, a proteinase secreted by macrophages and regulating extracellular matrix degradation, is associated with fibrosis, indicating the therapeutic efficacy of thMSC-EVs in fibrosis [52, 53]. In vivo, thMSC-EVs significantly reduced the levels of TNF-𝛼, IL-6, and CCL-2 in *E. coli*-induced mice, with non-significant decreases in the mean of IL-1𝛼 and IL-1β (Fig. 7). Statistical non-significance can be attributed to the lack of antimicrobial effects of thMSC-EVs, though not measured in this study. In our previous study using LPS-preconditioned MSCs, TLR4 signaling activation mediated immune modulation and bacterial clearance synergistically, resulting in a broader anti-inflammatory response [31]. However, thMSC-EVs administration to our previous *E. coli*-induced meningitis rat model study did not show bacterial clearance, similar to the present study. The stimuli-dependent modulation of cargo in MSCs-derived EVs is a highly advantageous approach for developing disease-specific therapeutics [18]. In summary, this study confirmed the protective therapeutic effect of thMSC-EVs by significantly reducing alveolar wall thickening, congestion, vascular permeability, and macrophage activation.

Challenges in the classical method of CT image analysis in small rodents include ambiguous air-surface contrast and complicated segmentation of the lung parenchymal regions because of intrathoracic structures, including the vasculature, heart, and airways [54, 55]. Here, we present a more feasible and accurate method for quantitative CT image analysis of small rodents by manually but precisely segmenting the lungs using semi-automatically calculated aerated parenchymal volumes. The therapeutic effect of thMSC-EVs was further confirmed macroscopically using CT image analysis. Quantitative CT image analysis revealed reduced diffuse patchy regions after intratracheal thMSC-EVs administration. Thus, we present a reliable and reproducible CT image analysis method which allows the precise evaluation of lung tissue in small rodents.

Despite several recent studies, the demand for an effective treatment for ARDS/ALI remains unmet [56–58]. A pharmacological approach investigates the use of drugs like anticoagulants, utilizing the effective targeting of a specific signaling pathway. A study suggested that nebulized antithrombin effectively ameliorated acute lung injury by decreasing coagulation and inflammation without altering the systemic coagulation [59]. MSCs are another promising therapeutic approach for lung injury, considering their stable secretion and delivery of regenerative factors to neighboring cells via EVs [13, 60–62]. WJ-MSCs are known for their higher proliferative capacity, non-tumorigenic properties, and rich secretome than those with other sources of MSCs [13, 63]. Recently, MSCs-derived EVs have been clinically evaluated for treating ARDS. [64–68]. EVs are advanced stem cell therapeutics, characteristically cell-free, low in immunogenicity and tumorigenic potential, and are more feasible form as “off the shelf” therapeutics [69–71]. Studies on preconditioned MSCs or derived EVs in ARDS/ALI preclinical models are not frequently performed despite their significant advantage in enhancing therapeutic efficacy. Therefore, the significance of this study lies in its investigation of the therapeutic efficacy of cargo-enhanced, injury-preexposed MSCs in an ALI experimental model. Considering that the therapeutic effect of EVs depends on their cargo, strategies to modulate and enhance its cargo cannot be overemphasized as the power of EV therapeutics. In that sense, thMSC-EVs may offer a broader range of therapeutic effects than specific pathway-targeting therapeutics such as anticoagulants in ARDS, since its much-enhanced cargo and production amount secreted by the MSCs with thrombin preconditioning involve various pathways but not limited to anti-inflammation. During the isolation of thMSC-EVs using the TFF system, thrombin should have theoretically filtered out, leaving no significant leftovers to alter the study results. However for translational research, the confirmation of thrombin leftover levels will be included in future studies.

From gross to microscopic analysis, we have thoroughly evaluated the therapeutic effect of thMSC-EVs in reducing *E.coli*-induced acute lung injury. thMSC-EVs reduced tissue damage, pulmonary edema, inflammatory cytokine levels (CCL2, TNF-α, and IL-6), and damaged lung regions on CT image analysis, which are crucial therapeutic indicators of ARDS/ALI. There was no significant difference in body weight and mortality, which are indicators of animal well-being. However, we postulate that considering the severity of ALI-induction and intratracheal, not systemic, administration of thMSC-EVs, 3-day observation was too short to observe acute improvements in body weight and survival. A longer observation may help confirm the holistic animal improvements. Therefore, a long-term follow-up study will be needed. In conclusion, therapeutically enhanced MSC-EVs represent a novel potential therapeutic option for ARDS/ALI.

### Electronic supplementary material

Below is the link to the electronic supplementary material.

Supplementary Material 1

Supplementary Material 2

Supplementary Material 3

## Data Availability

All data generated or analyzed during this study are included in this published article and its supplementary information files.
