# Supplementary material for: Therapeutic efficacy of thrombin-preconditioned mesenchymal stromal cell-derived extracellular vesicles on Escherichia coli-induced acute lung injury in mice
Source: Respir Res. 2024 Aug 7;25:303. doi: 10.1186/s12931-024-02908-w (PMC11308396; doi:10.1186/s12931-024-02908-w)
Supplement: Supplementary file 1 — Supplementary Material 1 [file 12931_2024_2908_MOESM1_ESM.docx]

**Supplementary material**


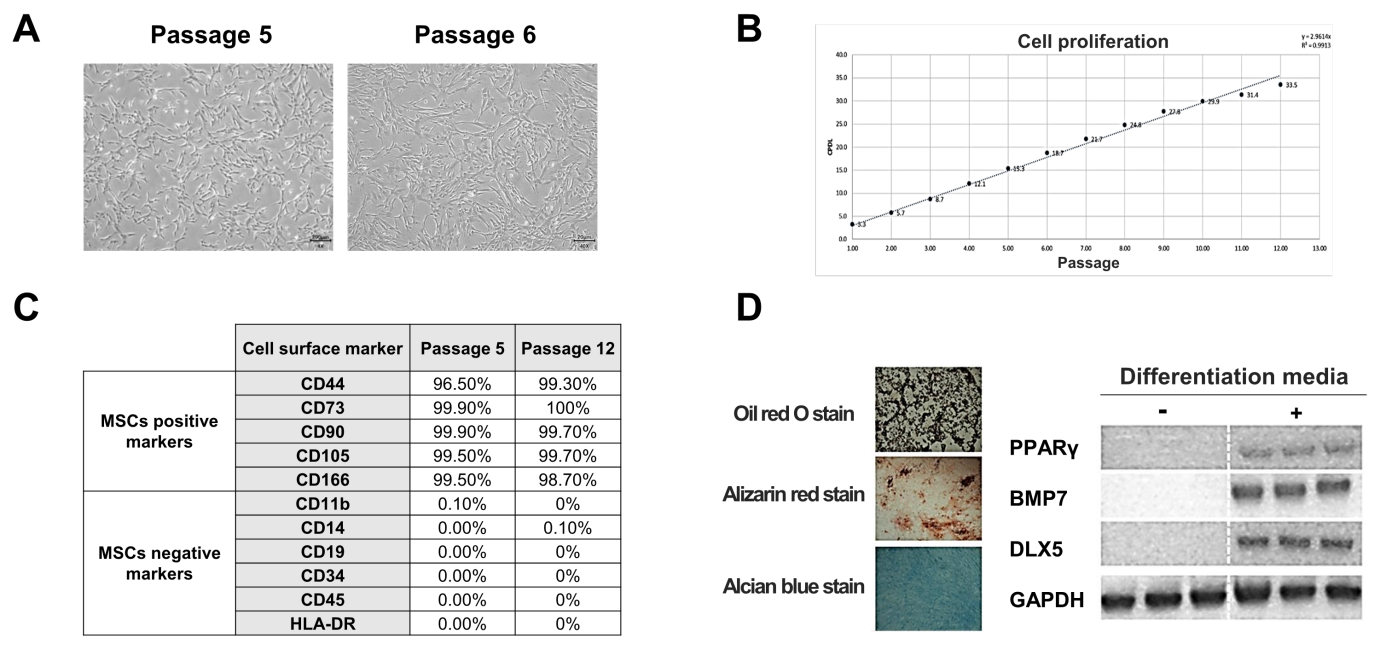


**Figure S1. Characterization of WJ-MSCs at passage 6. (A)** Morphological characteristic of spindle-shaped WJ-MSCs. **(B)** Sustained proliferation rate of WJ-MSCs from passage 1 to 12. **(C)** Confirmation of surface markers of WJ-MSCs using FACS. **(D)** Confirmation of multipotent differentiation potential of WJ-MSCs. Differentiated adipocytes, osteocytes, and chondrocytes were confirmed by staining with Oil Red O, Alizarin Red, and Alcian Blue, respectively.


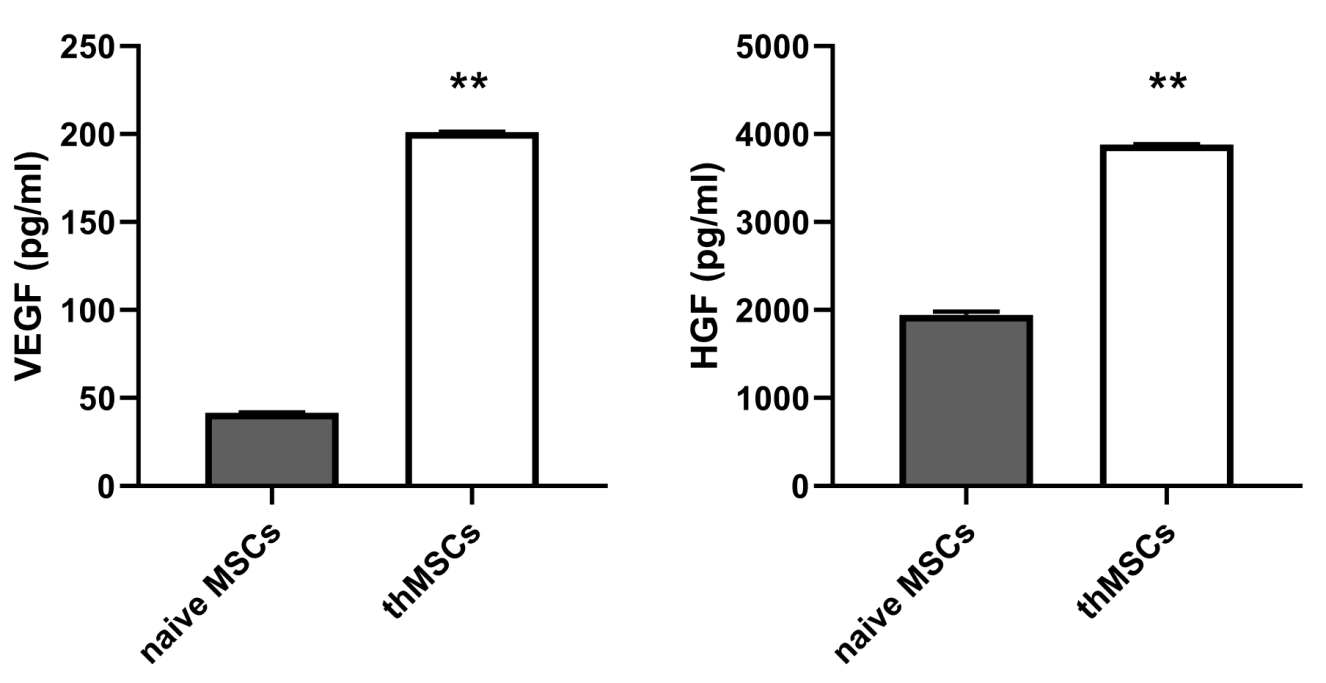


**Figure S2. Confirmation of increased levels of VEGF and HGF in thrombin-preconditioned MSCs.** Bar graph representation of VEGF and HGF measured from naïve and thMSCs’ conditioned medium. Data are expressed as m$\mathrm{ean}\pm$ standard error of mean (SEM). **, *p* <0.01 vs. naïve MSCs group. An unpaired t-test was used. thMSCs, thrombin preconditioned MSCs.


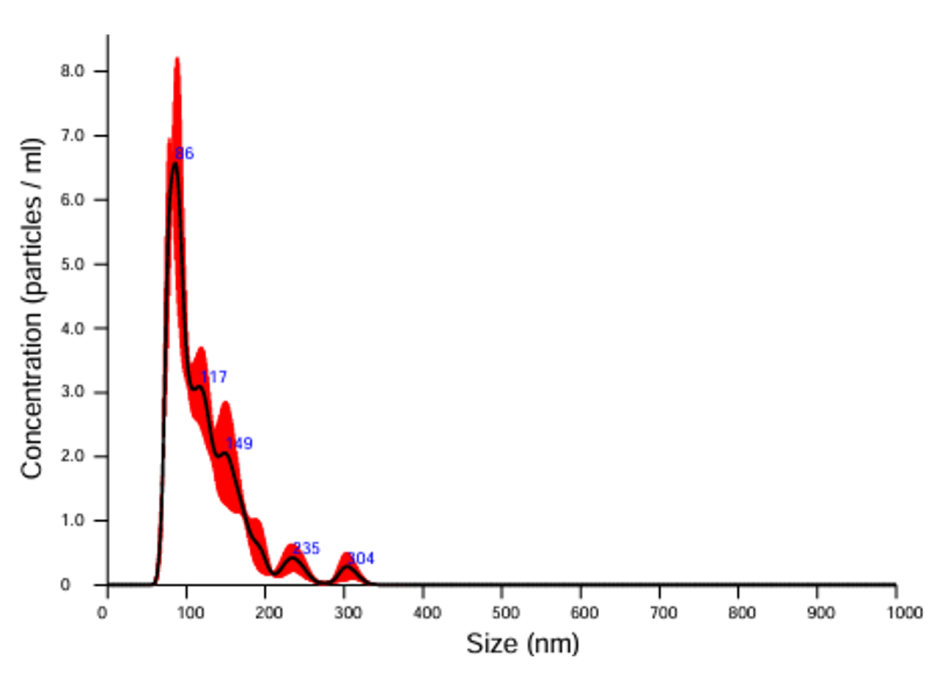


**Figure S3. Nanoparticle tracking analysis (NTA) result of naïve MSC-EVs.**


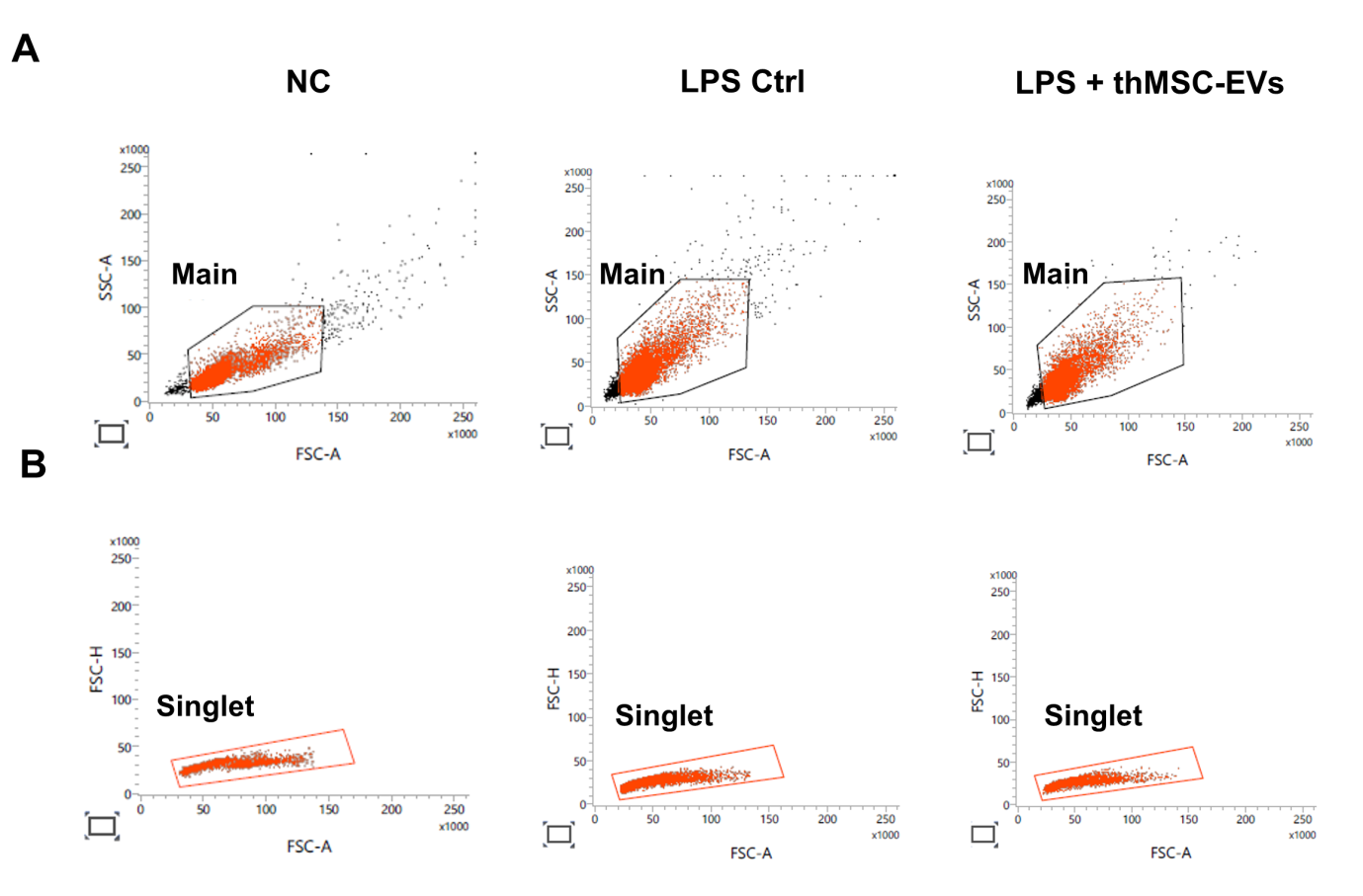


**Figure S4. Gating strategy used to analyze LPS-induced RAW264.7 cells. (A)** Exclusion of debris and dead cells. **(B)** Selection of singlets.


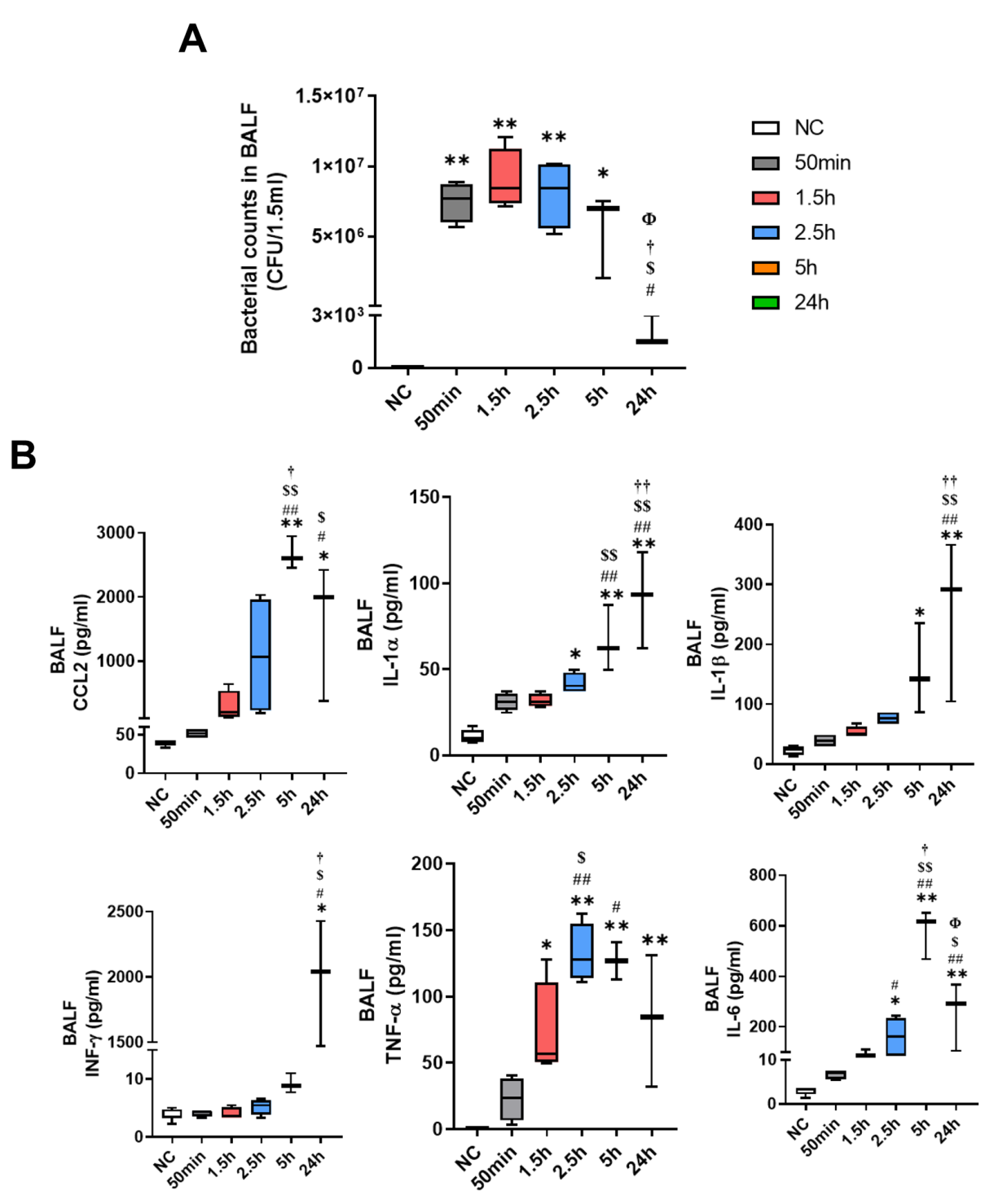


**Figure S5. Time-course measurement of bacterial CFU and pro-inflammatory cytokines in BALF.** **(A)** Measured bacterial CFU from BALF. **(B)** The levels of CCL-2, IL-1α, IL-1β, INF-γ, TNF-α, and IL-6 were measured using ELISA. Data are represented as a box and whisker plot. Whiskers represent the min and max. *, *p* <0.05 vs. NC; #, *p* <0.05 vs. 50 min.; $, *p* <0.05 vs. 1.5 h; †, *p*<0.05 vs. 2.5 h; Φ, *p*<0.05 vs. 5 h. One-way ANOVA post hoc Tukey was used. The x-axis represents the time after bacterial inoculation. n = 5, 4, 4, 4, 3, 3 in NC, 50 min, 1.5 h, 2.5 h, 5 h, and 24 h groups, respectively. NC, normal control; CFU, colony forming units.


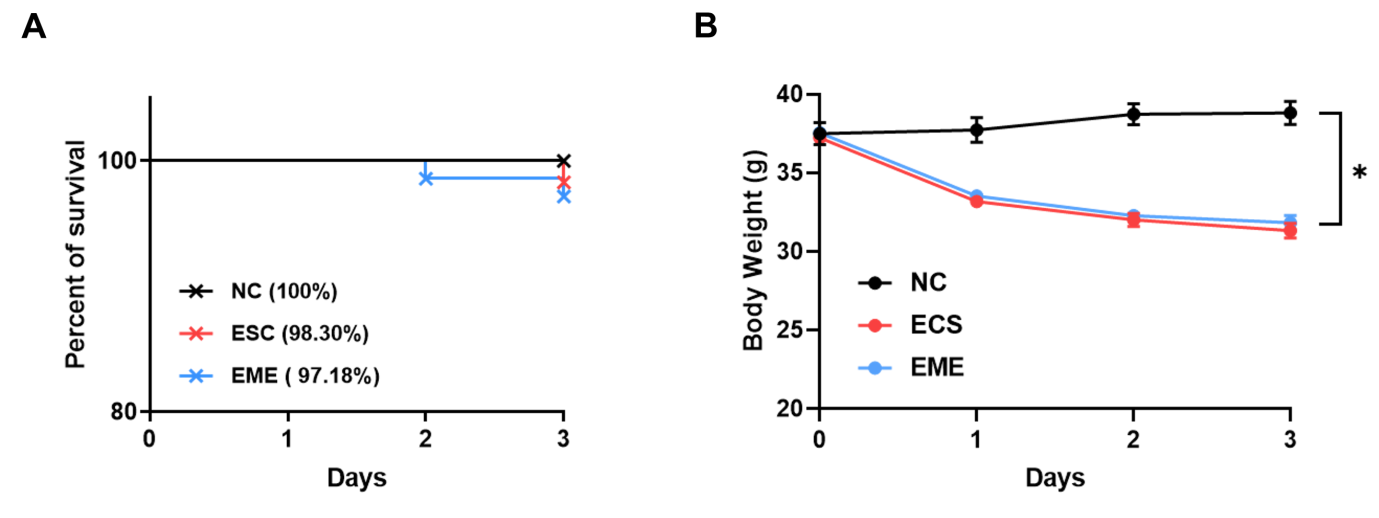
 **Figure S6.** **Survival rates and body weights of ALI in vivo model**. **(A)** The survival rate of NC (n=34; 100%), ECS (n=59-1; 98.30%), and EME (n=71-2; 97.18%) were measured using log-rank test. The survival rates were not significantly different among all groups. **(B)** Daily body weights of NC, ECS, and EME groups. Data are expressed as m$\mathrm{ean}\pm$ standard error of mean (SEM). *, *p* <0.05 vs. NC. One-way ANOVA post hoc Tukey analysis was used. NC, normal control; ECS, *E. coli*-induced ALI control group; EME, thMSC-EVs treatment group after *E. coli*-induced ALI.
