# Supplementary material for: Therapeutic efficacy of thrombin-preconditioned mesenchymal stromal cell-derived extracellular vesicles on Escherichia coli-induced acute lung injury in mice
Source: Respir Res. 2024 Aug 7;25:303. doi: 10.1186/s12931-024-02908-w (PMC11308396; doi:10.1186/s12931-024-02908-w)
Supplement: Supplementary file 2 — Supplementary Material 2 [file 12931_2024_2908_MOESM2_ESM.docx]

| **Supplementary Table 1:** Bacterial CFU cultured from BALF. | | | | | | | | |  |  |  |
| --- | --- | --- | --- | --- | --- | --- | --- | --- | --- | --- | --- |
|  |  |  |  |  |  |  |  |  |  | |  |
|  | **Percentage of mouse tested positive for *E. coli*** | | |  | **Bacterial CFU/mL** | | | | |  | |
|  |  |  |  |  |  |  |  |  |  |  | |
| **Group** | **Positive** | **%** | **Total** |  | **Min** | **Max** | **Median** | **MEAN ± SEM** | |  | |
| **NC** | 0 | 0 | 27 |  |  |  |  |  | |  | |
| **ECS** | 9 | 29.03 | 31 |  | 10 | 3350 | 10 | 476.7 ± 365.5 | |  | |
| **EME** | 10 | 31.25 | 32 |  | 10 | 1170 | 15 | 282.0 ± 125.4 | |  | |

NC, normal control; ECS, *E. coli-induced* ALI control group; EME, thMSC-EVs treatment group after *E. coli*-induced ALI.
