# Supplementary material for: Therapeutic efficacy of thrombin-preconditioned mesenchymal stromal cell-derived extracellular vesicles on Escherichia coli-induced acute lung injury in mice
Source: Respir Res. 2024 Aug 7;25:303. doi: 10.1186/s12931-024-02908-w (PMC11308396; doi:10.1186/s12931-024-02908-w)
Supplement: Supplementary file 3 — Supplementary Material 3 [file 12931_2024_2908_MOESM3_ESM.docx]

**Therapeutic efficacy of thrombin-preconditioned mesenchymal stromal cell-derived extracellular vesicles on *Escherichia coli*-induced acute lung injury in mice**

^1,4†^Yuna Bang, ^1,2†^Sein Hwang, ^1,3^Young Eun Kim, ^1^Dong Kyung Sung, ^1,3^Misun Yang, ^1,3^So Yoon Ahn, ^1,3^Se In Sung, ^2,4^Kyeung Min Joo, ^1,2,3*^Yun Sil Chang

**Supplementary File**

| **Supplementary Table 2:** Lung scan imaging parameters used in the study. | | |
| --- | --- | --- |
| **Acquisition parameters** | **Specification** |  |
| Voltage | 80 kV |  |
| X-ray source current | 400 ㎂ |  |
| Exposure time | 400 ms |  |
| Effective pixel size (resolution) | 39.77 ㎛ |  |
| Aluminum filter thickness | 1.5 mm |  |
| Slice thickness | 0.079547 mm |  |
| Step size | 1º |  |
| Total rotation | 360º |  |
| Rotation steps | 360 |  |
| Scanning duration | 20 min |  |

**Detailed instructions for obtaining the semi-automatic air ROI value.**

1. Open the CT image in the IRW program and set the FOV to 2.68 × 4.05 cm.
2. Designate the lung area to obtain air ROI using the 'Shape tool' (Figure S7A).
3. Set the Threshold Range as -1000 to -150 HU and apply (Figure S7B).

** The range of HU can be determined by the experimental conditions applied.

1. When an undesired area is included (pink, Figure S7C), remove it using the ‘Eraser tool.’
2. The air ROI is generated with a unit of mm^3^ (Figure S7D).

**
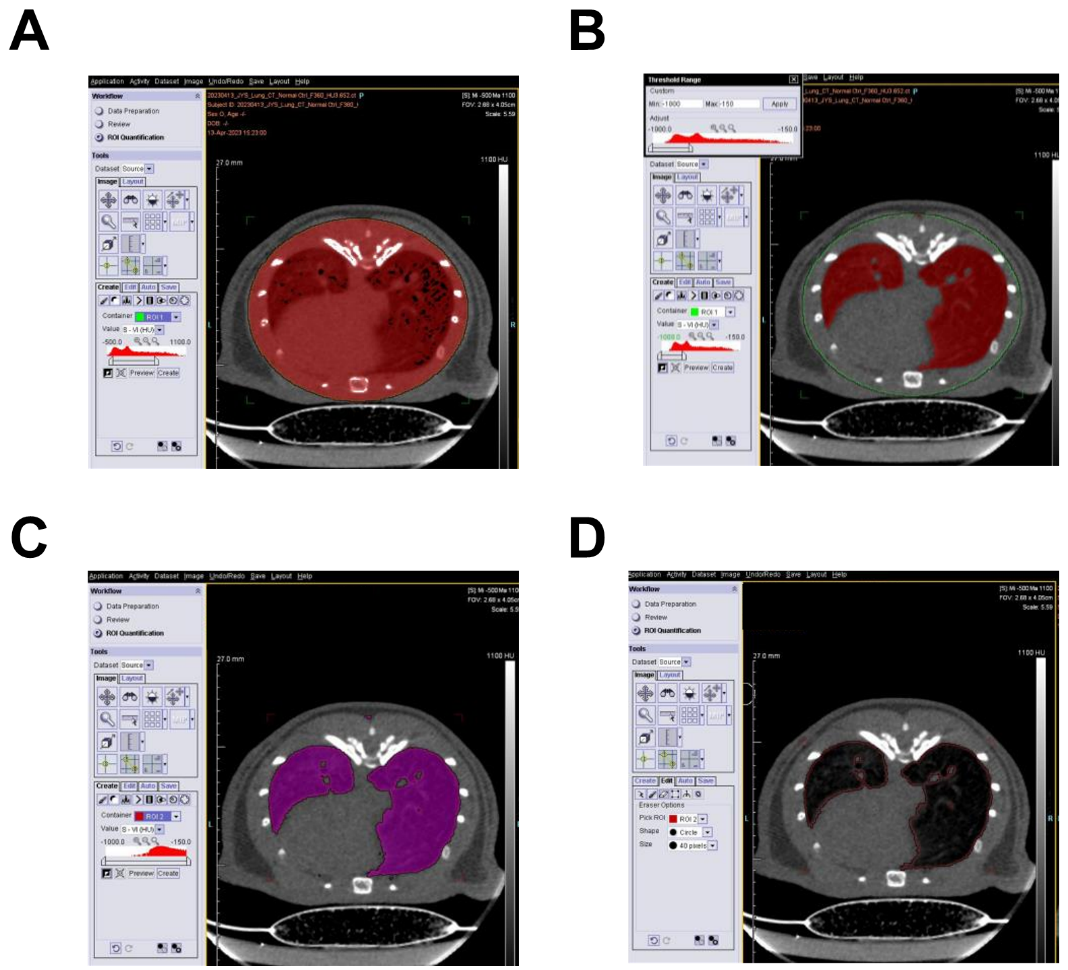
**

**Figure S7**. **Step-wise instruction for obtaining semi-automated lung air region of interest (ROI) measurements using the IAW program.** **(A)** Selection of desired lung tissue area for analysis. **(B)** Selected aerated region after threshold range applied. **(C)** Selection of non-aerated region to be erased. **(D)** The final image of obtained air ROI values from a single CT image. CT, computed tomography; FOV, field of view; HU, Hounsfield unit.

**Detailed instructions on how to work with manual ROI**

Important considerations before starting the following steps: To ensure no variation between the semi-automated air ROI measurement and manual lung contour ROI, the CT image must have a scale bar to calibrate in Image J program.

1. Open the CT image with a 2.68 × 4.05 cm FOV in Image J and calibrate the image based on the scale bar of the image. Select the ‘Straight’ tool to measure the length of a scale bar to get the result.
2. Click ‘Set Scale’ on the ‘Analyze’ tab of the ImageJ program panel.
3. Set ‘distance in pixels’ as the measured length of the scale bar from step 2. Set pixel aspect ratio to 1.0. Set the ‘unit of length’ as mm.
4. Manually outline the lung as in Figure S8B. Tablet (Wacom; Kazo, Saitama, Japan) was used in this study with the unit set to mm^2^.
5. The lung outline volume is calculated by multiplying the area from Step 4 by the thickness of the CT image.
6. Repeat steps 1-5 independently to all selected CT images.
7.
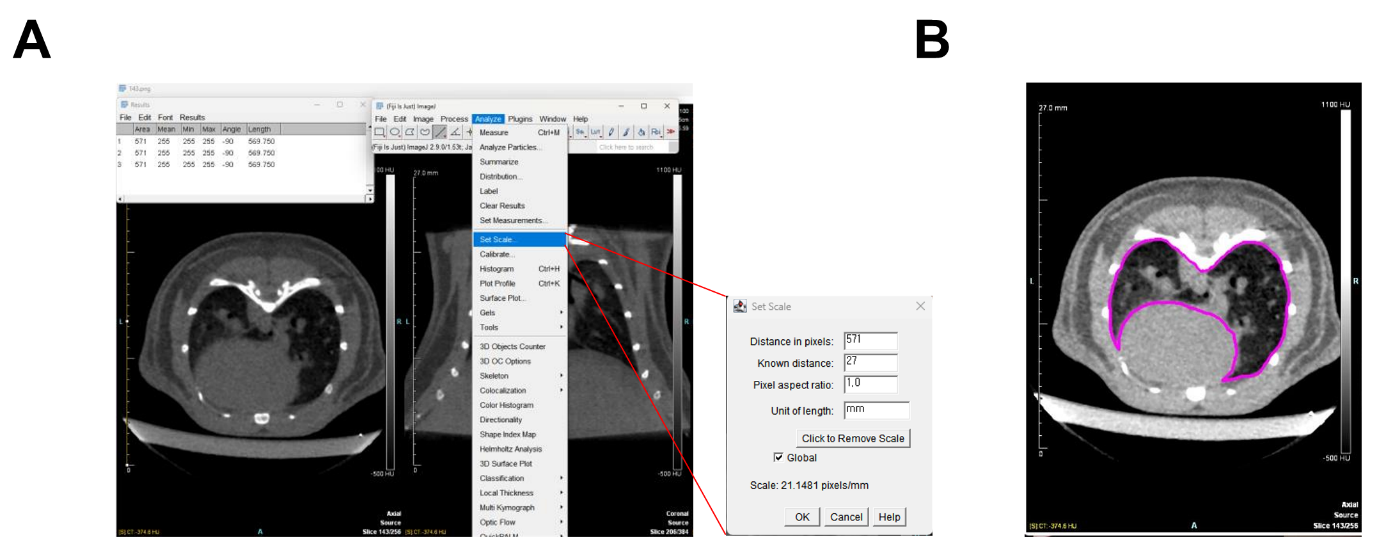
Calculate the percent tissue volume by subtracting the semi-automatically obtained air ROI value (Figure S7D) from the lung volume measured from step 6.

**Figure S8. A representative method of manual outlining of lung contour. (A)** Image calibration in the Image J program. **(B)** Manually outlined lung contour.


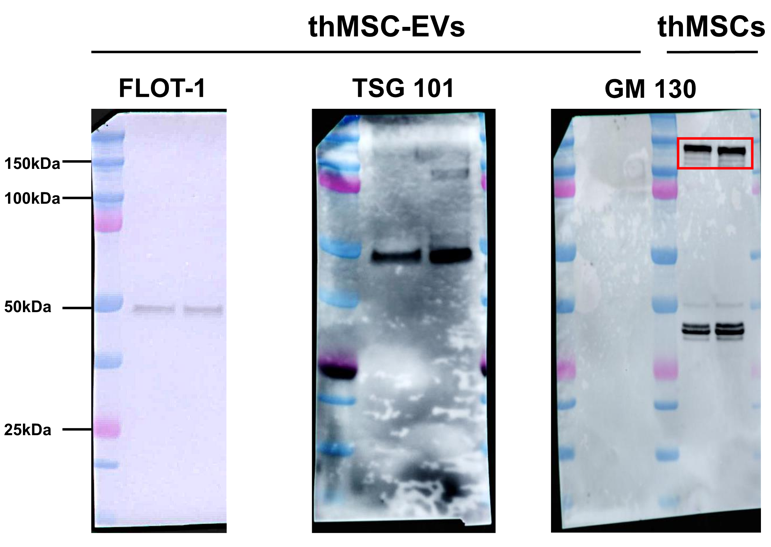


**Figure S9**. Full western blot images of EV-specific markers. EV-positive markers: FLOT-1 and TSG 101. EV-negative marker: GM 130 measured from (left lanes) thMSC-EVs and (right lanes) thMSCs. FLOT-1, Flotillin-1 (49kDa); TGS 101, Tumor susceptibility gene 101 (49kDa); GM130, Golgi matrix protein 130 (140kDa).
